# Alterations in Brain Structure and Functional Connectivity in Alcohol Dependent Patients and Possible Association with Impulsivity

**DOI:** 10.1371/journal.pone.0161956

**Published:** 2016-08-30

**Authors:** Junkai Wang, Yunli Fan, Yue Dong, Mengying Ma, Yi Ma, Yuru Dong, Yajuan Niu, Yin Jiang, Hong Wang, Zhiyan Wang, Liuzhen Wu, Hongqiang Sun, Cailian Cui

**Affiliations:** 1 Neuroscience Research Institute, Peking University, Beijing, China; 2 Department of Neurobiology, School of Basic Medical Sciences, Peking University, Beijing, China; 3 Key Laboratory of Neuroscience, The Ministry of Education and Ministry of Public Health, Beijing, China; 4 Beijing Hui-Long-Guan Hospital, Peking University, Beijing, China; 5 Peking University Sixth Hospital/Institute of Mental Health and Key Laboratory of Mental Health, Ministry of Health, Peking University, Beijing, China; 6 Department of Magnetic Resonance, General Hospital of Armed Police Forces, Beijing, China; 7 Stereotactic and Functional Neurosurgery Laboratory, Beijing Neurosurgical Institute, Capital Medical University, Beijing 100050, China; Institute of Psychology, Chinese Academy of Sciences, CHINA

## Abstract

**Background:**

Previous studies have documented that heightened impulsivity likely contributes to the development and maintenance of alcohol use disorders. However, there is still a lack of studies that comprehensively detected the brain changes associated with abnormal impulsivity in alcohol addicts. This study was designed to investigate the alterations in brain structure and functional connectivity associated with abnormal impulsivity in alcohol dependent patients.

**Methods:**

Brain structural and functional magnetic resonance imaging data as well as impulsive behavior data were collected from 20 alcohol dependent patients and 20 age- and sex-matched healthy controls respectively. Voxel-based morphometry was used to investigate the differences of grey matter volume, and tract-based spatial statistics was used to detect abnormal white matter regions between alcohol dependent patients and healthy controls. The alterations in resting-state functional connectivity in alcohol dependent patients were examined using selected brain areas with gray matter deficits as seed regions.

**Results:**

Compared with healthy controls, alcohol dependent patients had significantly reduced gray matter volume in the mesocorticolimbic system including the dorsal posterior cingulate cortex, the dorsal anterior cingulate cortex, the medial prefrontal cortex, the orbitofrontal cortex and the putamen, decreased fractional anisotropy in the regions connecting the damaged grey matter areas driven by higher radial diffusivity value in the same areas and decreased resting-state functional connectivity within the reward network. Moreover, the gray matter volume of the left medial prefrontal cortex exhibited negative correlations with various impulse indices.

**Conclusions:**

These findings suggest that chronic alcohol dependence could cause a complex neural changes linked to abnormal impulsivity.

## Introduction

Abnormal impulsivity is repeatedly mentioned to link with several psychiatric disorders including substance addiction according to diagnostic and statistical manual of mental disorders, 4th edition (DSM-IV) [1]. There is extensive evidence indicating that heightened impulsivity likely contributes to the development and maintenance of alcohol and other substance use disorders [2, 3]. Understanding the abnormal impulsivity related neural changes in alcohol dependent patients (ADPs) is very important to improve the treatment outcome in the clinical practice. However, there is still a lack of neuroimaging studies that comprehensively examined the brain changes associated with abnormal impulsivity in the context of alcohol dependence. According to previous literature, impulsivity may be defined as ‘‘a predisposition toward rapid, unplanned reactions to internal or external stimuli without regard to the negative consequences of these reactions [4].” Some other definition of impulsivity also related impulsivity to risk taking, lack of planning and making up one’s mind quickly [5]. Taken together, these literatures suggest that impulsivity is multi-dimensional construct, and that different aspects of impulsivity may reflect separate processes [6]. Linking to the drug use and dependence, the most commonly identified processes are behavioral inhibition, impulsive decision making and risky taking. The most commonly used behavioral measures of impulsivity are delay discounting task, which assesses impulsive decision making, Go/No-Go task, which assesses behavioral inhibition, and balloon analogue risk task, which assesses risky taking [7].

Numerous studies have proved that ADPs usually display higher impulsivity using questionnaire of impulsivity like Barratt impulsivity scale (BIS [8]) [9], as well as worse performance in different behavioral tasks like delay discounting task [10], Go/No-Go task [11] and balloon analogue risk task [12]. Neuroimaging studies also have revealed some brain structural changes associated with higher impulsivity assessed by different behavioral measures in ADPs [13, 14]. These structural abnormalities mainly existed in the orbitofrontal cortex (OFC), the medial prefrontal cortex (mPFC), the anterior cingulate cortex (ACC), the amygdala and the striatum [15, 16]. Up to now, very few studies focused on the relationship between white matter (WM) integrity and impulsivity in ADPs.

To date, most imaging studies reported brain functional changes associated with abnormal impulsivity in ADPs usually focusing on the activated or inhibited brain areas during subjects performing impulsive tasks. A variety of brain regions were thought to be involved in this process, including the OFC, the mPFC, the ACC, the striatum and the nucleus accumbens [17, 18, 19]. These brain areas are regarded as key nodes of the mesocorticolimbic system which is the most important circuitry involved in drug addiction and substantially influences goal-directed behaviors [20, 21]. However, drug addiction is considered alterations in circuit-level interactions between brain regions [20, 21]. Therefore, more and more studies tend to investigate the functional connectivity using functional magnetic resonance imaging (fMRI), largely because it allows for the exploration of large-scale networks and their interactions, thus moving towards a systematic level understanding of brain function [22]. Furthermore, resting-state functional connectivity (rsFC) has emerged as a powerful tool to identify neural circuitry dysfunction of various psychiatric disorders, including substance addiction [22, 23]. Thus far, there is little information about the relationship between rsFC and abnormal impulsivity in ADPs. Only one clinical study reported that the impulsivity measures were significantly correlated with altered rsFC in salience network (SN), default mode network (DMN) and left executive control network (LECN) [24]. Moreover, a few studies revealed the relationship between the frontostriatal dysfunction and impulsivity in other forms of addicted individuals. Specifically, compared with healthy conrtols, methamphetamine-dependent subjects displayed lower striatal D(2)/D(3) receptor availability and a significant negative relationship between impulsivity and dopamine D(2)/D(3) receptor availability in the striatum [25]. Structural and functional abnormalities in the frontostriatal circuits were also reported to associate with aspects of impulsivity in cocaine dependence [26, 27]. In summary, based on the existing data, we speculated that ADPs would have brain structural and functional abnormalities in the mesocorticolimbic system and these brain abnormalities would be correlated with various impulse indices.

The present study was designed to investigate the structural and functional connectivity changes in the brain associated with abnormal impulsivity in ADPs. To address this issue, differences in gray matter volume (GMV) and WM integrity across the whole brain between ADPs and HCs were first examined. Then, brain areas that existed GMV deficits and were associated with impulsivity based on aforementioned evidence were selected as seed regions for rs-fcMRI analysis to determine the dysfunction of impulsivity-related network. Finally, the correlative analysis between aberrant brain structure, functional connectivity and impulse behavior from ADPs was performed to reveal the relationships among them.

## Materials and Methods

### Participants

A total of 20 abstinent alcohol dependent right-handed male patients and 20 age-matched male healthy controls (HCs) participated in this study. All ADPs were recruited from Beijing Hui-Long-Guan hospital. The HCs were recruited from the local community. All participants were financially compensated for their participation. The experimental procedure was approved by the research ethics committee of Beijing Hui-Long-Guan hospital. Written informed consent was obtained from all participants after the study had been fully explained.

All ADPs were diagnosed as alcohol dependence according to DSM-IV criteria by psychiatrists. A Chinese version mini international neuropsychiatric interview was performed to exclude other Axis I psychiatric disorders in ADPs and HCs [28]. The severity of alcoholism was assessed using Michigan alcohol screening test (MAST [29]). ADPs had no previous substance dependence or current substance abuse other than alcohol and nicotine. Additional exclusion criteria for ADPs and HCs were as follows: 1) A history of head injury with loss of consciousness; 2) The presence of any past or current neurological disease; 3) Presence of hepatitis C, hypertension and type 2 diabetes that required medical intervention; 4) Age above 60 years and below 19 years; and 5) The presence of any contraindications to an MRI. Demographic, clinical and behavioral data were analyzed with SPSS 11.5 using 2-sample *t* tests or Chi-square test. All results were summarized in Table 1.

**Table 1:** Demographic, behavioral data and clinical characteristics of subjects.

Characteristic	HCs	ADPs	*P* value
Participants (n)	20	20	─
Age	40.50 ± 8.17	43.95 ± 6.30	0.14
Years of education	9.15 ± 4.18	11.60 ± 2.70	**0.034**
Age of onset of drinking	─	18.70 ± 2.68	─
Average dose of lifetime (number of drinks per day)	0.33 ± 0.31	15.31 ± 11.21	**< 0.001**
Dose during peak use (number of drinks per day)	0.33 ± 0.31	25.40 ± 14.51	**< 0.001**
Length of abstinence until MRI session	─	41.5 ± 10.80	─
Cigarette smokers (no. of smokers)	14	19	0.096
Current cigarettes per day	19.56 ± 10.28	22.42 ± 12.97	0.48
Years of smoking cigarettes	23.17 ± 5.17	21.52 ± 7.79	0.58
MAST scores^**a**^	0.90 ± 2.05	31.50 ± 4.61	**< 0.001**
BIS-11 Total impulsiveness scores ^**b**^	57.06 ± 4.06	66.90 ± 7.54	**< 0.001**
BIS-11 Nonplanning scores^**b**^	25.25 ± 2.53	28.00 ± 4.76	**0.028**
BIS-11 Motor impulsiveness scores^**b**^	20.40 ± 2.46	22.70 ± 3.47	**0.020**
BIS-11 Attentional impulsiveness scores^**b**^	11.95 ± 1.93	16.20 ± 3.16	**< 0.001**
Go/no-go task			
Errors of commission (n)	1.30 ± 1.56	1.70 ± 2.39	0.53
Errors of omission (n)	0.50 ± 0.95	0.95 ± 1.36	0.23
Reaction time (ms)	432.60 ± 42.52	446.69 ± 61.72	0.41
BART task			
Average adjusted pumps (n)	37.89 ± 18.81	57.68 ± 17.39	**< 0.01**

Note: Mean and standard deviation for each group; t-tests or Chi-square test were applied to test for group differences; statistical significance level was set at *P* < 0.05 (two-tailed). Significant *P*-values were in bold.

^**a**^MAST, Michigan Alcoholism Screening Test (Selzer ML 1971)

^**b**^ BIS-11, Barratt Impulsiveness Scale 11th version (Patton JH et al. 1995).

Before the experiment, ADPs have been detoxified on a ward. All patients were abstinent for at least 1 month (mean: 41.5 days) and free of psychoactive medications for at least 1 week before behavioral tests and magnetic resonance imaging (MRI) scans. Each participant attended behavioral tests and MRI scans in two separate days within 1 week.

### Questionnaire and neuropsychological measures

#### Barratt impulsiveness scale

Impulsivity was assessed by the Chinese version of the Barratt impulsiveness Scale, 11th edition (BIS-11), a validated questionnaire that measures impulsiveness in three main dimensions [8]. Three subscales are attention impulsiveness, motor impulsiveness and non-planning impulsiveness. This scale contains thirty 4-point Likert-type items. Items are rated from 1 (rarely/never) to 4 (almost always/always). The BIS scores of subjects were listed in Table 1.

#### Delay discounting task (DDT)

A computerized version of the delay discounting task, as described by [30] was adapted for the current study. Briefly, the task presented a series of hypothetical choices between a standard monetary amount available after a delay and a variable monetary amount available immediately. The standard item was ¥10.00 available after a delay of 0, 7, 30, 90, 180, or 365 days. The immediate option was an amount of money (ranging from ¥0 to ¥10.50 and further amounts increasing in 0.50 increments up to ¥10.50) available after 0 days. Standard items and variable immediate options were presented in a random order to make up a total of 138 questions. Subjects were asked to choose one option between the standard larger delayed option and the variable smaller immediate option they preferred for each question.

For each participant, an indifference point was determined for each of six delays. The indifference point is the value of the variable immediate reward at which the preference switches from the delayed to the variable immediate reward. The rate of delay discounting, also known as k value, was determined by fitting the 6 indifference points to the hyperbolic equation: V = A/ (1 + k*D) for each participant. Besides, the delay discounting task was considered unsuccessful if the participant chose the same option on ≥85% of the trials (e.g., always or nearly always chose the delayed reward or the immediate reward on trials in which two options were individualized to be equally attractive) [31].

#### Go/No-Go task

In this study, a simple Go/No-Go paradigm, as described by [32] was programmed by using E-Prime v1.1 experiment generation software (Psychology Software Tools, Inc., Pittsburgh, PA, USA). A trial was consisted of five main events: 1) 800 ms fixation; 2) 500 ms blank white screen; 3) a cue, displayed a blank white arrow presented in either a horizontal or vertical orientation, and required no response. The display time was randomized with intervals between 100ms and 500ms; 4) followed by a Go or No-Go condition, visible until a response occurred or 1000 ms elapsed; and 5) 1000 ms intertrial interval. A horizontal cue is followed by a Go condition 80% of the time and a vertical cue is followed by a No-Go condition 80% of the time. Participants were instructed to respond by pressing the space bar as quickly as possible when the screen appeared the blue arrow (Go signal) and to not respond when the screen appeared the green arrow (No-Go signal). A total of 200 trials were presented lasting approximately 10 minutes. The main dependent variables were commission errors (responses to the No-Go signal), omission errors (failure to respond to the Go signal) and reaction time.

#### Balloon analogue risk task (BART)

A computerized version of balloon analogue risk task, as described by [33] was also programmed by using E-Prime v1.1 experiment generation software. In the BART, participants were told they would be presented with 30 virtual balloons on the computer screen and they could inflate balloon by clicking the left mouse button. Each button press can add ¥0.05 to a temporary bank. Each balloon was set to burst after an unpredictable number of pumps and at this point all the money in the temporary account would be lost. The participant was free to stop pumping at any point before the balloon burst by clicking the right mouse button. Then, the current money could be collected from the temporary bank and added to a permanently safe bank. Participants were encouraged to make as much money as possible. The dependent measure of this task was the average number of pumps on all unexploded balloon trials during the performance (the adjusted number of pumps) [33].

### MRI data acquisition

All imaging data were acquired on a Siemens 3 Tesla Trio scanner by using the standard whole head coil. We used three different MRI techniques to investigate brain changes between ADPs and HCs: 1) a high resolution T1-weighted image to measure GMV; 2) a blood oxygenation level-dependent (BOLD) functional imaging was acquired to assess rsFC of the brain; and 3) a diffusion tensor imaging was collected to assess the integrity of white matter (WM). The details of the imaging parameters were as follows:

Whole brain high-resolution T1-weighted images were acquired by using a multiecho magnetization prepared rapid gradient echo (MPRAGE) sequence (repetition time = 2300ms, echo time = 2.98ms, flip angle = 9°, acquisition matrix = 240 × 256, field of view = 256 mm ×256 mm, 176 slices, voxel size = 1 ×1 × 1 mm^3^). Blood oxygenation level-dependent (BOLD) functional imaging was conducted using a T2*-weighted single-shot, gradient-recalled echo planar imaging (EPI) sequence (repetition time = 2000 ms, echo time = 30 ms, flip angle = 90°, acquisition matrix = 64 × 64, field of view = 256 mm × 256 mm, slice thickness = 4 mm, gap = 1 mm, voxel size = 3.91 × 3.91 × 5 mm^3^, 29 axial slices, 180 image volumes). The diffusion tensor imaging (DTI) was collected using a single-shot, spin-echo echo-planar imaging sequence with alignment of the anterior-posterior commissural plane (repetition time = 6100 ms, echo time = 93 ms, flip angle = 90°, acquisition matrix = 128 × 128, field of view = 256 mm × 256 mm, slice thickness = 3 mm, slices = 45). The diffusion sensitizing gradients were applied along 30 non-collinear gradient encoding directions with b = 1000 s/mm^2^ and the b = 0 s/mm^2^ experiment repeated four times.

### Data processing and analysis

We used the voxel-based morphology (VBM) technique to investigate GMV changes over the whole brain in ADPs. VBM analysis was conducted using SPM8 (Wellcome Department of Imaging Neuroscience, London, UK; http://www.fil.ion.ucl.ac.uk/spm). Briefly, the following steps were performed on the T1 images. MR images were first segmented into GM, WM and cerebrospinal fluid (CSF). Then, the GM and WM partitions of each subject in the native space were high dimensionally registered and normalized to the standard Montreal Neurological Institute(MNI) space using diffeomorphic anatomical registration through exponentiated lie algebra (DARTEL) normalization as implemented in the SPM8. After normalization, the GM images with modulation were smoothed with a Gaussian filter of 8 mm full-width half-maximum kernel. Next, we conducted voxel-by-voxel comparisons of GMV between ADPs and HCs using a two tailed two-sample *t*-test with age, years of education and total brain GMV as covariates. A voxel level threshold was set at *P* < 0.05 (FWE-corrected). For further exploring the relationships between abnormal GMV and behavioral measures, 10-mm sphere masks were defined around the peak voxels of the significant GM clusters. The relevant ROI signals were extracted using REST version 1.8 ([34]; http://restfmri.net/forum/REST_V1.8).

Resting state fMRI (rsfMRI) images were analyzed by using SPM8 and REST version 1.8 implemented in Matlab 7.11 (MathWorks Inc., Natick, MA, USA). Prior to pre-processing, the first 10 volumes were discarded to allow the magnetization to approach a steady state. The remaining data were preprocessed with slice timing correction, motion correction (realignment). One patient and one healthy subject were excluded because of excessive head motion (more than 2 mm translation or 2° rotation).Then, the data were normalized to standard MNI space and smoothed by a Gaussian filter with a full width half maximum (FWHM) of 8 mm. Subsequently, the data were temporally filtered with a band-pass filter (0.01–0.08 Hz), followed by removal of the linear trend. Nine nuisance signals were further regressed out by using averaged signals from WM, CSF and the whole brain plus six parameters obtained from head motion correction. Functional connectivity was measured by using a seed-based approach by choosing bilateral dACC, right dPCC, left mPFC, right OFC and left putamen cluster peaks as the center of the ROI (radius = 6 mm) from VBM analysis in MNI template (see Table 2 for exact location). The cross-correlation coefficients between these seed voxels and all other voxels were calculated to generate correlation maps. These correlation maps were converted to Z-value maps using Fisher’s r-to-z transformation to improve the normality of the correlation coefficients. Next, group analyses were performed for the correlation maps of each seed region. A Voxelwise two-sample *t*-test was performed to compare the correlation maps derived from each seed between ADPs and HCs. Significance level was set at cluster level FWE corrected threshold of *P* < 0.05.

**Table 2:** Grey matter volume reduction in ADPs versus HCs (FWE corrected for multiple comparisons across the entire volume).

Regional clusters	Side	Cluster size	Peak MNI coordinates			Maximal Z-value
			X	Y	Z	
Dorsal posterior cingulate cortex	R	1829	2	-19	49	5.76
Dorsal anterior cingulate cortex	L	1829	-3	20	33	5.65
	R	1829	3	33	25	5.56
Medial prefrontal cortex	L	1829	0	15	51	5.55
Premotor cortex	R	1829	3	-1	55	4.89
Primary somatic sensory cortex	L	432	-51	-13	45	5.57
Orbitofrontal cortex	R	271	5	36	-14	5.43
Precuneus	L	189	-5	-67	10	5.22
Putamen	L	121	-30	12	3	5.13
Inferior frontal gyrus	L	42	-48	11	25	5.05
Cerebellum	R	500	6	-66	-41	5.40

Note: R = Right, L = left. MNI: Montreal Neurological Institute.

DTI data were analyzed using the FMRIB Diffusion Toolbox from the FSL processing software package (http://www.fmrib.ox.ac.uk/fsl) and a pipeline toolbox, PANDA ([35]; http://www.nitrc.org/projects/panda/). Briefly, the DTI images were first checked for visible quality issues and one healthy subject was excluded due to severe motion. Then, the raw data were skull-stripped. Next, the data were corrected for motion and eddy current distortions using affine transformation to the b0 image. The diffusion tensor for each voxel was then estimated by the multivariate linear fitting algorithm, and the output generated voxel-wise maps of fractional anisotropy (FA), axial diffusivity (AD) (λ1), radial diffusivity (RD) (average of λ2 and λ3) and mean diffusivity (MD) (average of λ1, λ2, and λ3) for each participant. Voxel-wise whole brain analysis of FA images was performed by using tract-based spatial statistics (TBSS) [36]. The FA maps generated for each participant were transformed from individual space to a standard Montreal Neurological Institute (MNI) space via spatial normalization. Then, the registered FA images were averaged to obtain a mean FA image. The mean FA image was applied to create a mean FA skeleton. The skeleton was then thresholded at a value of 0.2 to exclude non-white matter tissue. After this, each subjects aligned FA image was projected onto the mean FA skeleton to create a skeletonized FA map. To identify FA differences between groups, the skeletonized FA data were fed into the voxel-wise statistical analysis. The testing was based on non- parametric approach and performed by the FSL randomize program, which uses 5000 random permutations [37]. Age and educational levels were entered into the analysis as covariates. The results were considered significant for *P*<0.05 corrected for multiple comparisons using the Threshold-Free Cluster Enhancement (TFCE) method [38]. The most probable anatomic localization of each significant cluster was determined using the JHU-ICBM-DTI-81 white matter labels atlas in MNI space. For clusters where significantly lower FA values in ADPs were observed, the mean AD, RD and MD values of each cluster mask for each subject were extracted. The mean values of the diffusivity indices were calculated by the function “fslmeants” from FSL.

Two sample t tests were applied to test for group differences on demographic, clinical characteristics and behavioral measures except for number of smokers which was performed using Chi-square test. Last, Pearson’s correlations were used to investigate the relationships among aberrant GMV, functional connectivity, WM integrity and impulsive indices including BIS-11 total scores, (ln) k value and average adjusted pumps in the ADPs. The results were threshold at *P* < 0.05 Bonferroni-corrected for multiple comparisons.

## Results

### Demographic characteristics and behavioral measures

All subjects underwent impulse-related behavioral tests and MRI scans. There were no significant differences in age and smoking status between ADPs and HCs, except for the difference in years of education (*P* < 0.05). ADPs showed a severity of alcohol dependence (on the MAST: *P* < 0.001) and obviously greater amount of lifetime alcohol intake (*P* < 0.001. See Table 1) compared with HCs.

**Fig 1:**
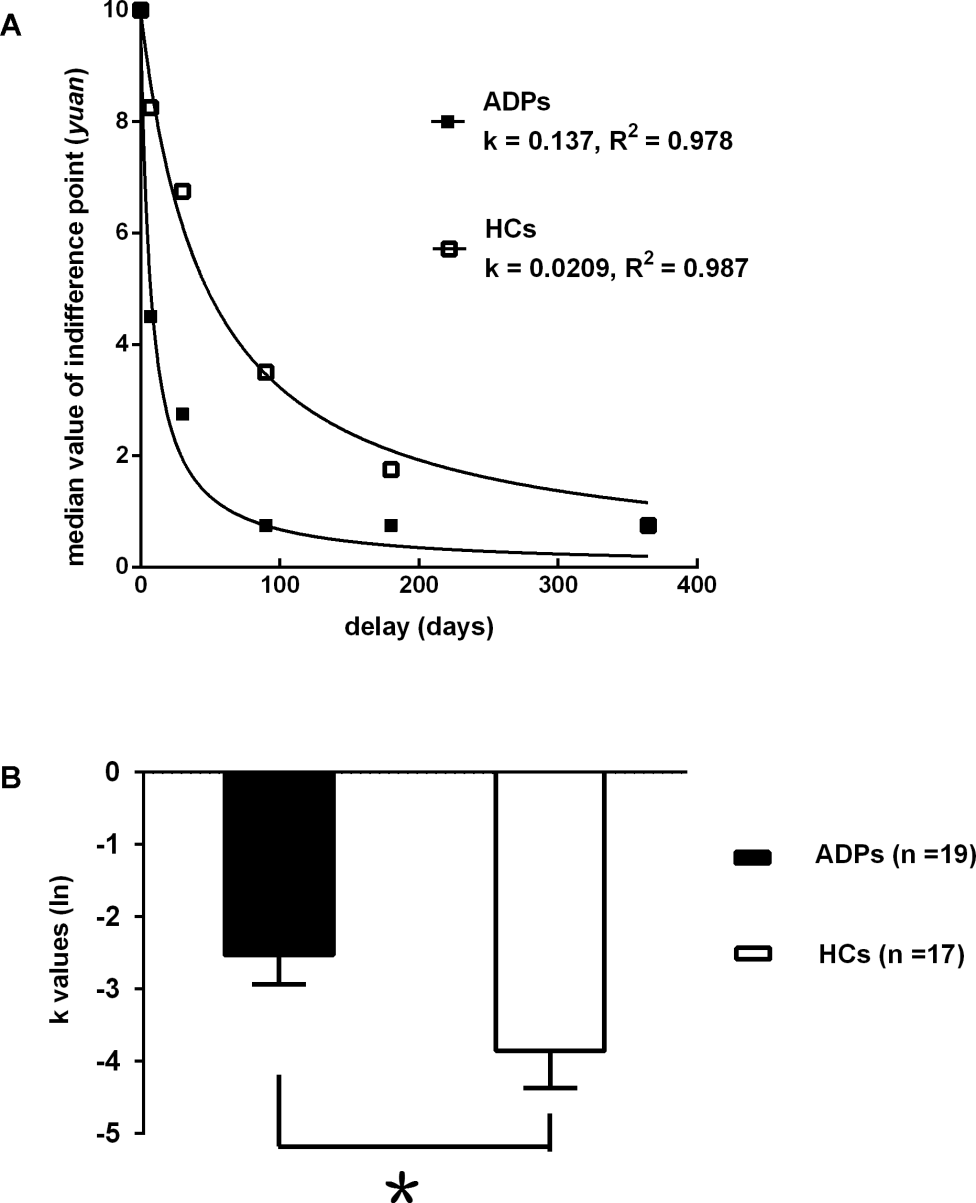
(A) Delay discounting in ADPs and HCs. Data points represent the median indifference points at each time delay. The lines reflect the best fit of the hyperbolic function V = 10/(1+k*D) to the median group indifference points. The rate of discounting for each group (k value) and the goodness of fit for both function are also shown. (B) Mean (ln) k-values when the hyperbolic function is fitted to the data for each subject.Smaller (ln) k-values indicate greater discounting. Err bars represent SEM. The distribution of k values was extremely positively skewed and leptokurtic and the group variances were significantly heterogeneous. Therefore, a natural logarithm (ln) transformation was computed before performing the t test analyses. *p < 0.05 compared with HCs.

In contrast to HCs, ADPs had higher scores on the total score of the BIS-11 (*P* < 0.001) and in terms of the attentional impulsiveness (*P* < 0.001), non-planning impulsiveness (*P* < 0.05) and motor impulsiveness (*P* < 0.05) factors of the BIS-11(see Table 1). In the Go/No-Go task performance, there were no significant differences in errors of commission (*P* = 0.53), errors of omission (*P* = 0.23) and reaction time (*P* = 0.41) between two groups (see Table 1). In the DDT performance, ADPs showed significantly larger (ln) k values than HCs (*P* < 0.05. See Fig 1A and 1B). ADPs also showed significantly higher average adjusted pumps than HCs in the BART performance (*P* < 0.01. See Table 1).

### MRI results

**Fig 2:**
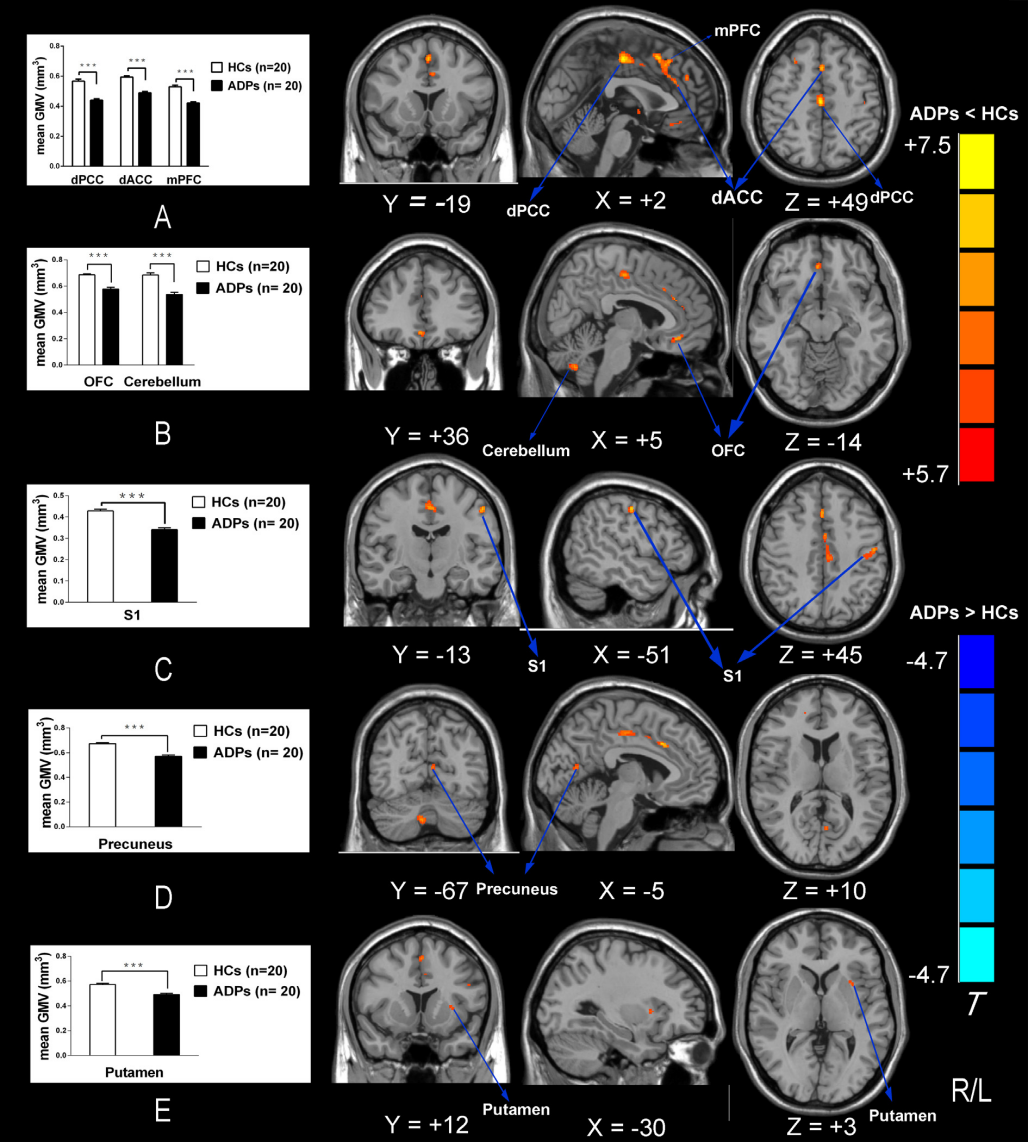
Whole-brain GMV was compared between ADPs and HCs using SPM8 plus DAREL analysis. VBM differences reflect GMV decreased in ADPs including the bilateral dACC, the left dPCC, the left mPFC, the right premotor cortex, the left S1, the right OFC, the left precuneus, the left putamen and the right cerebellum. (A-E) GMV was also extracted from the regions that revealed significantly lower GMV in ADPs shown in the Fig Abbreviations: dACC, dorsal anterior cingulate cortex; dPCC, dorsal posterior cingulate cortex; mPFC, medial prefrontal cortex; S1, primary somatic sensory cortex; OFC, orbitofrontal cortex; GMV, grey matter volume; ADPs, alcohol dependent patients; HCs, healthy controls.

ADPs showed a significant lower global GMV than HCs (ADPs: 583.95 ± 50.26 ml; HCs: 627.87 ± 50.13 ml; *P* < 0.01), as well as increased CSF volume (ADPs: 286.79 ± 33.46 ml; HCs: 208.31 ± 21.11 ml; *P* < 0.001). There was no significant difference in the total volume of WM between two groups (ADPs: 500.54 ± 58.15 ml; HCs: 532.34 ± 42.37 ml; *P* > 0.05). VBM analysis showed the most significantly reduced GMV in ADPs in the right dPCC and the bilateral dACC extending to the left mPFC and the right premotor cortex. Moreover, we also found significantly reduced GMV in ADPs in the right OFC, the left primary somatic sensory cortex, the left precuneus, the left putamen and the right cerebellum (see Table 2 and Fig 2). No significant increases in GMV were found in ADPs compared with HCs.

**Fig 3:**
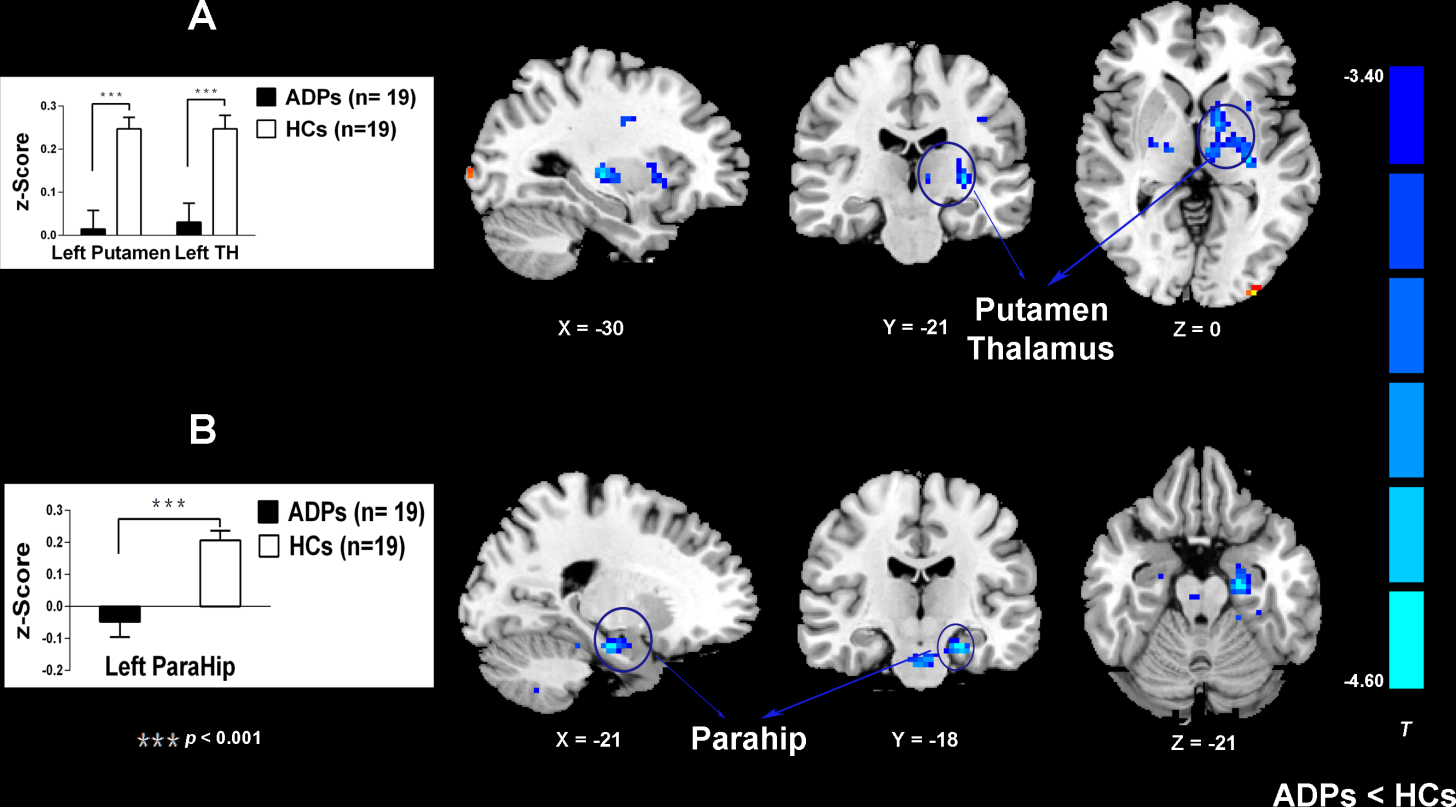
Group differences of rsFC existed between the seeds and the cluster location. Brain regions showed group differences include the thalamus, the putamen and the ParaHip. (A, B) Mean rsFC z scores were shown for ADPs and HCs. The ADPs showed weaker functional connectivity in reward network. Abbreviations: TH, thalamus; ParaHip, parahippocampal gyrus; ADPs, alcohol dependent patients; HCs, healthy controls.

Following these observed structural alterations in ADPs, we then conducted the rsFC analysis. Brain areas exhibiting altered rsFC with each seed ROI in ADPs were shown in Fig 3 and Table 3. Compared with HCs, ADPs exhibited reduced rsFC in multi-regions. Specifically, the left mPFC seed showed lower connectivity with the left putamen and the left thalamus in ADPs compared with HCs. The right OFC seed also demonstrated lower connectivity with the left parahippocampal gyrus in ADPs compared with HCs.

**Table 3:** Group differences of rsFC between the seeds and the cluster location (*p* < 0.05, cluster level FWE corrected).

Seeds	Regional clusters	Side	Cluster size	Peak MNI coordinates			Maximal Z-value
				X	Y	Z	
**Left mPFC**							
HCs > ADPs	Putamen	L	189	-30	-21	0	4.54
	Thalamus	L	189	-9	-15	3	4.17
**Right OFC**							
HCs > ADPs	ParaHip	L	104	-21	-18	-21	4.35

Note: mPFC, medial prefrontal cortex; OFC, orbitofrontal cortex; ParaHip, parahippocampal gyrus; ADPs, alcohol dependent patients; HCs, healthy controls; R = Right; L = left; MNI: Montreal Neurological Institute.

**Fig 4:**
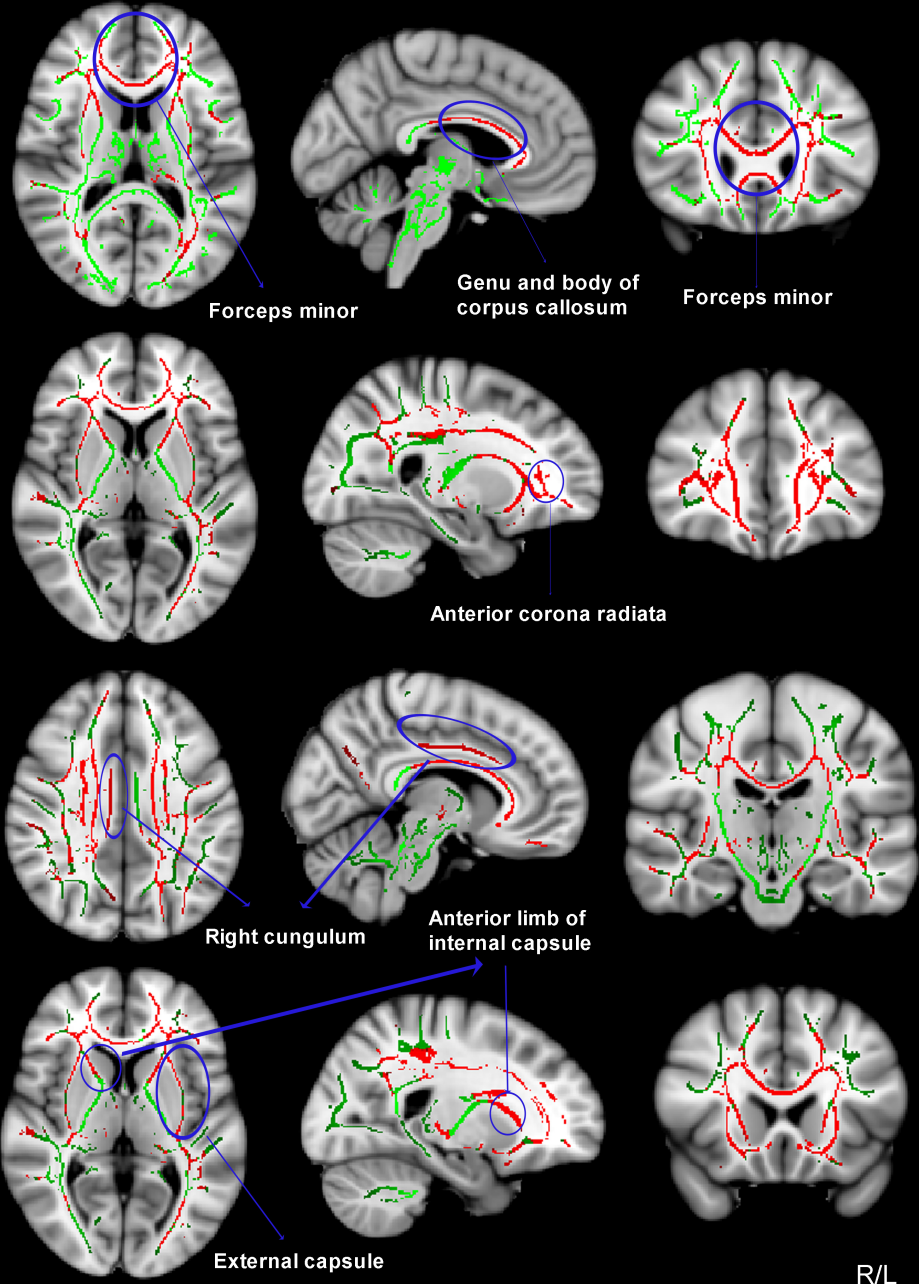
TBSS analysis of FA reduction (red) in ADPs versus HCs (*p* < 0.01, corrected by TFCE). Results are overlaid on MNI152-T1 template and the mean FA skeleton (green). The left of the image corresponds to the right hemisphere of the brain.

We also compared between-group differences in the WM integrity using TBSS analysis. Nearly 99.5% of the significant voxels localized to clusters 1 which contained all abnormal areas. Compared to HCs, ADPs showed significantly decreased FA in the bilateral anterior corpus callosum, the bilateral forceps minor, the bilateral anterior corona radiata, the right cingulum, the bilateral anterior limb of internal capsule and the bilateral external capsule (peak MNI (78, 172, 55), cluster size = 58505, *P* < 0.05 corrected. See Fig 4 and Table 4). There were no WM regions where ADPs had significantly higher FA than HCs. Additionally, we conducted multiple comparisons of MD, RD and AD of abnormal areas between two groups. We found that ADPs had significantly higher RD value than HCs in all areas, whereas differences between AD values were not significant between two groups. (see Table 4).

**Table 4:** Mean (± SD) values of DTI parameters in the cluster in which decreased FA was observed in TBSS analysis in ADPs versus HCs (*p* < 0.01, TFCE corrected).

White matter tract and location		ADPs (n = 20)	HCs (n = 19)	*P* value
Genu and body of corpus callosum (bilateral frontal lobe)				
	FA	0.51 ± 0.048	0.61 ± 0.052	**< 0.001**
	AD	1.34 ± 0.076	1.35 ± 0.076	0.78
	RD	0.55 ± 0.069	0.44 ± 0.053	**< 0.001**
	MD	0.82 ± 0.064	0.75 ± 0.043	**< 0.001**
Bilateral forceps minor (bilateral frontal lobe)				
	FA	0.71 ± 0.030	0.76 ± 0.045	**< 0.001**
	AD	1.67 ± 0.11	1.62 ± 0.072	0.14
	RD	0.38 ± 0.060	0.31 ± 0.059	**< 0.001**
	MD	0.81 ± 0.071	0.75 ± 0.047	**0.002**
Bilateral anterior corona radiata (bilateral frontal lobe)				
	FA	0.46 ± 0.032	0.52 ± 0.039	**< 0.001**
	AD	1.23 ± 0.065	1.21 ± 0.063	0.48
	RD	0.58 ± 0.039	0.51 ± 0.048	**< 0.001**
	MD	0.80 ± 0.041	0.74 ± 0.044	**0.001**
Right cingulum (cingulate gyrus)				
	FA	0.48 ± 0.041	0.54 ± 0.063	**0.001**
	AD	1.15 ± 0.062	1.18 ± 0.057	0.13
	RD	0.55 ± 0.029	0.51 ± 0.043	**0.001**
	MD	0.75 ± 0.029	0.73 ± 0.033	0.13
Bilateral anterior limb of internal capsule(basal ganglia)				
	FA	0.47 ± 0.036	0.52 ± 0.041	**< 0.001**
	AD	1.20 ± 0.046	1.23 ± 0.061	0.11
	RD	0.54 ± 0.047	0.50 ± 0.041	**0.004**
	MD	0.76 ± 0.041	0.74 ± 0.037	0.12
Bilateral external capsule (basal ganglia)				
	FA	0.41 ± 0.025	0.46 ± 0.036	**< 0.001**
	AD	1.19 ± 0.054	1.17 ± 0.046	0.25
	RD	0.63 ± 0.036	0.56 ± 0.038	**< 0.001**
	MD	0.81 ± 0.037	0.76 ± 0.032	**< 0.001**

Note: Significant *P*-values are in bold. AD, RD, MD: × 10^−3^ mm^2^/s.

### Correlation results

**Fig 5:**
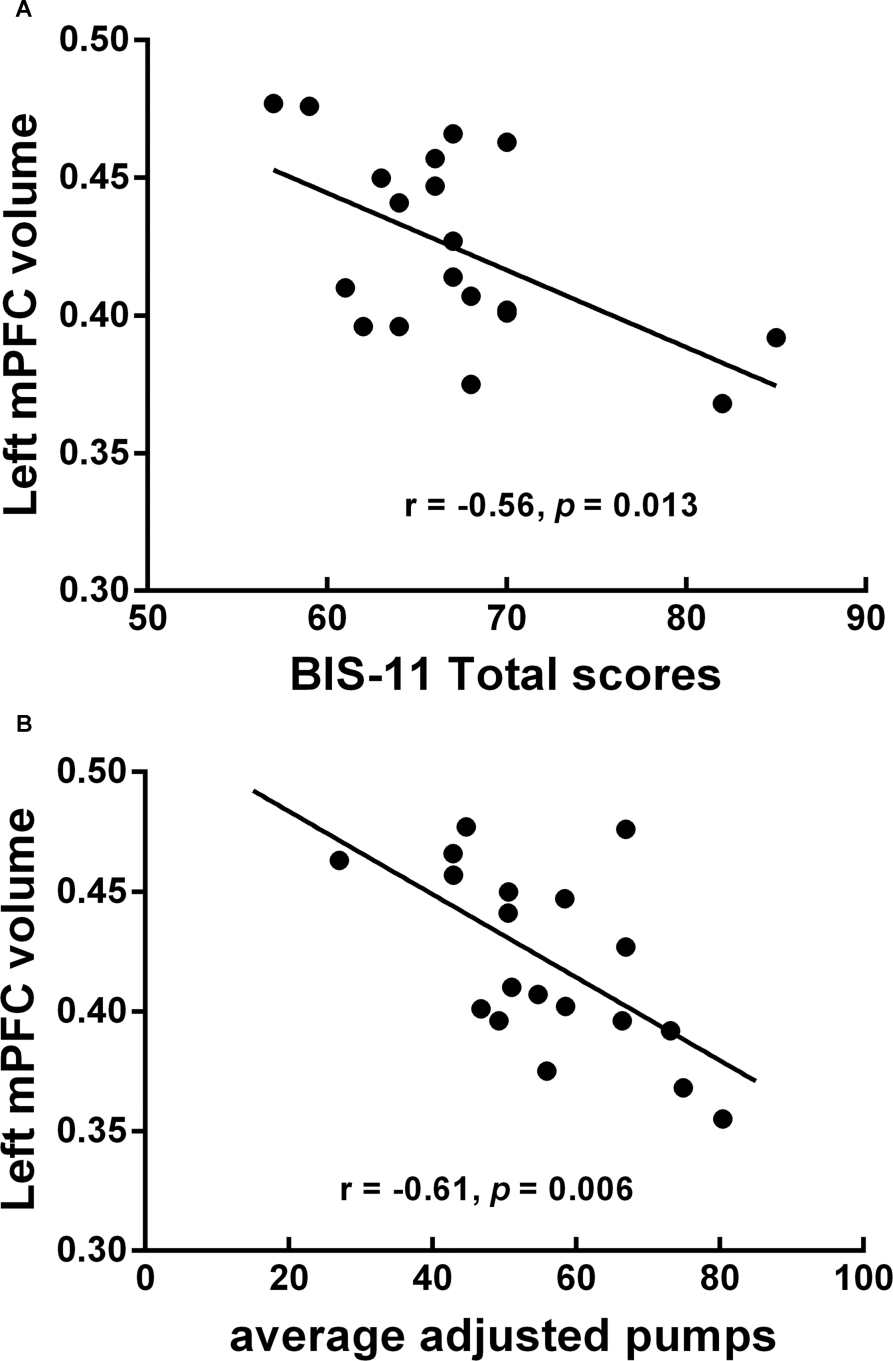
The GMV of the mPFC exhibited significant correlations with various impulsivity measures in ADPs. One patient’s BART score plus one patient’s BIS-11 scores were considered statistical outliers and excluded from correlation analysis. (A) Negative correlation between the total score of the BIS-11 and The GMV of the mPFC. (B) Negative correlation between average adjusted pumps and The GMV of the mPFC. Abbreviations: mPFC, medial prefrontal cortex; GMV, grey matter volume; BIS, Barratt Impulsiveness Scale; ADPs, alcohol dependent patients.

Our correlation analysis showed significant correlations between aberrant MRI indicators and behavioral measures in ADPs. Specifically, the GMV of the left mPFC, the left dACC and the left putamen exhibited negative correlation with various impulse indices. The total score of the BIS-11 showed negative correlation with the right cungulum’s FA. Average adjusted pumps showed positive correlation with the anterior corona radiate’s RD and negative correlation with the anterior corpus callosum and the anterior corona radiate’s FA. rsFC within the reward network was also negatively correlated with the total score of the BIS-11 and average adjusted pumps (see Table 5). However, most of these correlations didn’t survive correction for multiple comparisons. In fact, only the GMV of the left mPFC still exhibited significant negative correlation with the total score of the BIS-11 (r = -0.56, *P* **<** 0.05, Bonferroni correction; See Fig 5A) and average adjusted pumps (r = -0.61, *P* **<** 0.05, Bonferroni correction; See Fig 5B). Moreover, both correlations still showed significant after controlling for amount of alcohol intake per day.

**Table 5:** Brain-behavior correlations in ADPs.

Neuroimaging measures		Behavioral measures	Parametric model	Correlation Coefficient	*P* value
**Gray matter volume**					
Left dACC		BIS-11 Total scores	Linear	-0.50	**0.04**
Left mPFC		BIS-11 Total scores	Linear	-0.56	**0.013**
Left mPFC		(ln) K value	Linear	-0.65	**0.02**
Left dACC		average adjusted pumps	Linear	-0.51	**0.03**
Left putamen		average adjusted pumps	Linear	-0.49	**0.048**
Left mPFC		average adjusted pumps	Linear	-0.61	**0.006**
**DTI parameters**					
FA-Right cungulum		BIS-11 Total scores	Linear	-0.49	**0.048**
FA-Genu and body of corpus callosum		average adjusted pumps	Linear	-0.62	**0.02**
FA-Bilateral anterior corona radiate		average adjusted pumps	Linear	-0.59	**0.04**
RD-Bilateral anterior corona radiate		average adjusted pumps	Linear	0.56	**0.048**
**Functional connectivity**					
**Seed**	**Region**				
Left mPFC	left putamen	BIS-11 Total scores	Linear	-0.48	**0.04**
Left mPFC	left thalamus	average adjusted pumps	Linear	-0.66	**0.02**

Note: dACC, dorsal anterior cingulate cortex; mPFC, medial prefrontal cortex; ADP, alcohol dependent patients; HC, healthy controls; R = Right; L = left. Significant *P*-values are in bold.

## Discussion

In the current study, we found that ADPs displayed higher impulsivity in the BIS-11 and all three subscales as well as the performance of the DDT task and BART task. The MRI analysis revealed three main results: (1) ADPs had decreased GMV in the mesocorticolimbic system including the dPCC, the dACC, the mPFC, the OFC and the putamen; (2) ADPs showed significantly decreased FA in the anterior corpus callosum, the forceps minor, the anterior corona radiata, the cingulum, the anterior limb of internal capsule and the external capsule driven by higher RD value in these areas; and (3) rs-fcMRI analysis also revealed maladaptive interactions in the mesocorticolimbic system in ADPs. Additionally, correlation analyses indicated the GMV of the left mPFC exhibited significant correlations with various impulsivity measures.

In line with the previous data, our results also demonstrated that ADPs existed GM deficits in the dPCC, the dACC, the mPFC, the OFC and the putamen. These brain regions were closely associated with impulsivity as mentioned before [39, 40]. Given that impulsivity is multi-dimensional construct [7] and different aspects of impulsivity may reflect separate processes [6]. Therefore, these areas may serve as substrates and play distinct roles in different processes. The OFC was repeatedly mentioned to link impulsivity to drug addiction [41]. It was implicated in executive function and has emerged as a potential neural substrate to use the value of perceived or expected outcomes to guide decisions. Chronic drug use resulted in maladaptive changes in the OFC that were thought to disrupt the normal cognitive processes leading to poor decision making among drug addicts [41]. The mPFC was involved in cognitive control that monitored ongoing actions and performance outcomes and subsequent adjustments of behavior and learning to acquire flexible goal-directed behavior [42]. The deficits in this area may lead an impaired cognitive control over emotional and compulsive reactions [39]. Recent theories suggested that the ACC served as a monitoring role for the detection of erroneous or error-prone actions, together with the mPFC involved in the cognitive control [42, 43]. The putamen, a part of the striatum, has been proved as a core subcortical region involved in decision making and compulsive drug seeking and taking by numerous studies in substance addiction [20, 41]. The dPCC considered as key nodes of the DMN were also demonstrated to involve in decision making [44]. Our findings of the negative correlations between impulsivity measures and the GMV of the left mPFC confirmed this area was specially associated with impulsivity again. Unfortunately, we cannot directly address the causal relationships between these reductions, impulsivity and the severity of alcohol dependence due to the cross-sectional nature of the present study. However, this can be explained by neurotoxic effects of alcohol in the previous literature, in which chronic alcohol misuse could cause the reductions of these brain areas which heightened the impulsivity in ADPs. Both of them could aggravate the alcohol dependence. Taken together, we considered these areas as seed regions to determine the potential downstream effects of GMV reduction.

Another important finding of the current study was abnormal microstructural integrity of WM in ADPs accompanying with the GMV reduction. Lower FA has been suggested to reflect myelin loss and axonal damage [45]. Based on RD and AD measurements, the FA difference between the groups was driven by lower RD, indicating demyelination in ADPs. Our findings were also consistent with previous studies in alcohol dependence [46, 47]. The myelin abnormalities in patients might be explained by following factors: direct neurotoxic effects of alcohol, vitamin deficiency or genetic predisposition [47, 48]. Along with the GM deficits in ADPs, it was obvious to notice these abnormal WM regions just connected the damaged GM areas in ADPs. Moreover, the correlation results also showed negative trends between impulsivity measures and the cungulum, the anterior corpus callosum and the anterior corona radiata’s FA and positive trends between impulsivity measures and the anterior corona radiata’s RD. These findings also revealed the relationships between the impulsivity deficits and the abnormal microstructural integrity of WM in ADPs. Together, these findings may suggest abnormal WM integrity among the mesocorticolimbic system was associated with higher impulsivity in ADPs.

To determine the potential downstream effects of brain structure deficits in ADPs, we compared rsFC between two groups. The results revealed ADPs exhibited significantly decreased rsFC among several regions including the mPFC, the OFC, the putamen, the thalamus and the parahippocampal gyrus compared with HCs. These areas were considered as key nodes in reward network [49]. These decreased functional connectivities in ADPs may suggest impaired “top-down” cognitive control [50]. Chronic alcohol misuse had neurotoxic effects on ‘‘top-down” control regions such as the mPFC and the OFC and then may jeopardize their ability to control over subcortical areas like the putamen, the thalamus and the parahippocampal gyrus. In addition, the correlation results also suggested these decreased rsFC had negative correlation trends with impulsivity measures. Taken together, we proposed that individual needed fully functional reward network to maintain impulsivity at a normal level. Chronic alcohol dependence may impair the ability of reward network. So, ADPs had to increase the ability of executive control regions such as the mPFC and the OFC to achieve a better performance on cognitive control which may suggest processes of neuroadaptive plasticity.

Moreover, VBM analysis showed the most significantly reduced GMV in ADPs in the right dPCC and the bilateral dACC extending to the left mPFC and the right premotor cortex. We also found GMV reductions were more marked in the right hemisphere than in the left hemisphere. These findings were also supported by the previous proposition that right hemisphere functions may be more vulnerable than left hemisphere functions to the effects of chronic alcoholism [51, 52]. However, rsFC results in our study showed inconsistent findings, compared with HCs, ADPs exhibited significantly decreased rsFC in the left hemisphere. These results may be explained by the participants who were all right-handed recruited in this study. So, the executive control processes may tend to be more dominant in left hemisphere than the other. The lateralization of the results shown in this study may be influenced by the combined effects of the chronic alcohol misuse and the handedness.

Some limitations in this study should be considered. Most ADPs and HCs were smokers. We can’t rule out the potential effects of this confounding factor on the brain. However, the alcohol dependence may still be the dominant factor contributing to the brain abnormalities between ADPs and HCs according to previous study [53]. Next, educational level was different between two groups. However, this confounding factor didn’t interfere in the structural and functional brain changes observed in heroin-addicted subjects [54]. Besides, we also considered educational level as a covariate to avoid this effect on any observed differences in the structure and function of the brain between groups when we performed the MRI analysis. Finally, like most clinical studies, our study was a cross-sectional study. We can’t directly address the causal relationship between abnormal impulsivity and long-time alcohol dependence. In the future, we intend to perform a longitudinal study to explore the relationship between abnormal impulsivity and early relapse in ADPs.

Overall, in this study, we found GM deficits in the mesocorticolimbic system including the dPCC, the dACC, the mPFC, the OFC and the putamen accompanying with abnormal microstructural integrity of WM connecting these GM areas had downstream effects on rsFC of reward network in ADPs, which may contribute to higher impulsive behavior and underlie the neurobiological mechanism for impulsivity. In a word, these findings suggest that chronic alcohol dependence could cause a complex neural changes linked to abnormal impulsivity.
